# Evaluating mental workload during multitasking in simulated flight

**DOI:** 10.1002/brb3.2489

**Published:** 2022-03-15

**Authors:** Wenbin Li, Rong Li, Xiaoping Xie, Yaoming Chang

**Affiliations:** ^1^ Department of Aerospace Hygiene Faculty of Aerospace Medicine Air Force Medical University Xi'an Shaanxi P. R. China; ^2^ Department of Internal Medicine Faculty of Clinical Medicine Xi'an Medical University Xi'an Shaanxi P. R. China

**Keywords:** flight, fNIRS, heart rate variability, mental workload, multitasking

## Abstract

**Background:**

Pilots must process multiple streams of information simultaneously. Mental workload is one of the main issues in man–machine interactive mode when dealing with multiple tasks. This study aimed to combine functional near‐infrared spectroscopy (fNIRS) and electrocardiogram (ECG) to detect changes in mental workload during multitasking in a simulated flight.

**Methods:**

Twenty‐six participants performed three multitasking tasks at different mental workload levels. These mental workload levels were set by varying the number of subtasks. fNIRS and ECG signals were recorded during tasks. Participants filled in the national aeronautics and space administration task load index (NASA‐TLX) scale after each task. The effects of mental workload on scores of NASA‐TLX, performance of tasks, heart rate (HR), heart rate variability (HRV), and the prefrontal cortex (PFC) activation were analyzed.

**Results:**

Compared to multitasking in lower mental workload conditions, participants exhibited higher scores of NASA‐TLX, HR, and PFC activation when multitasking in high mental workload conditions. Their performance was worse during the high mental workload multitasking condition, as evidenced by the higher average tracking distance, smaller number of response times, and longer response time of the meter. The standard deviation of the RR intervals (SDNN) was negatively correlated with subjective mental workload in the low task load condition and PFC activation was positively correlated with HR and subjective mental workload in the medium task load condition.

**Conclusion:**

HR and PFC activation can be used to detect changes in mental workload during simulated flight multitasking tasks.

## Graphical abstract

This study combined subjective ratings, performance, fNIRS, and ECG to evaluate the mental workload of a simulated piloting multitasking task. In this study, the program of multitasking tasks abstractly simulated flight characteristics and different levels of cognitive demand were set by the number of subtasks completed at the same time. The present study found that the comprehensive evaluation method can be used to evaluate mental workload and the sensitivity of PFC activation is better than that of HRV.

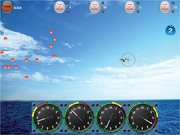

## 1 INTRODUCTION

With the development of aircraft automation and information technology, pilots have an increasing amount of information to process during flight. They often need to process information on multiple tasks at the same time and mental workload tends to be an issue in such cases (Hsu et al., 2015). It is a major psychological construct, describeable as the portion of limited mental capacity that is actually required to perform a particular task at the moment (O'Donnell & Eggemeier, 1986). Mental workload usually results from tasks requiring lower physical demand but higher demand on cognition, thinking, and judgment of the operator (Wickens et al., 2016). However, the cognitive resources of humans are fundamentally limited (Wickens, 2002). In some conditions, pilots must perform several tasks simultaneously, each with a different priority. The simultaneous appearance of multiple task information leads to a high mental workload (Hsu et al., 2015). In fact, high mental workload does not inherently have bad consequences, but high mental workload during multitasking can lead to less residual resources to perform concurrent tasks, which may result in task management errors (Wickens et al., 2016). In turn, task management errors may influence task switching. In that case, pilots may omit vital information due to the cognitive tunneling phenomenon, which was defined as “the allocation of attention to a particular channel of information, diagnostic hypothesis or task goal, for a duration that is longer than optimal, given the expected cost of neglecting events on other channels, failing to consider other hypotheses, or failing to perform other tasks” (Wickens, 2005). Many studies have highlighted that performance declines when cognitive demands exceed the operator's cognitive resources (Chenot et al., 2021; Fallahi et al., 2016; Puma et al., 2018; Stojan and Voelcker‐Rehage, 2021). Therefore, it is of theoretical and practical significance to evaluate the mental workload of pilots during multitasking. According to Dehais et al. (2020), the objective of measuring the mental workload of pilots during multitasking is to predict the probability of performance impairment.

Mental workload is an abstract attribute of man–machine interaction, which cannot be directly observed (Matthews et al., 2015). The evaluation of mental workload is generally conducted through various methods such as subjective reports, performance evaluations, and physiological measurements (O'Donnell & Eggemeier, 1986). One of the mostly used tools in the subjective report is the national aeronautics and space administration‐task load index (NASA‐TLX) scale (Hart, 2006). Performance evaluation can be divided into primary and secondary task performance evaluations. The primary task is the task that has the priority of processing when the operator needs to complete multiple tasks at the same time. In the case of priority completion of the primary task, the operator uses their residual capacity to complete the other task, the secondary task (Liu & Wickens, 1994). Methods of physiological measurement to evaluate mental workload include electroencephalogram (EEG), electrocardiogram (ECG), eye movement, and functional near‐infrared spectroscopy (fNIRS). Mental workload cannot be estimated precisely with a single index or method because individual and environmental factors will affect the mental effort deployed to perform a given task (Wanyan et al., 2014). Therefore, a comprehensive evaluation method is necessary.

Lehrer et al. (2010) combined heart rate variability (HRV), NASA‐TLX, and task performance to evaluate mental workload during a simulated flight with a Boeing 737 B flight‐800 Level D flight simulator. They found cardiac assessment to be a useful addition to self‐reported measures for determining flight task mental workload and risk for performance decrements. HRV is widely applied in the evaluation of mental workload because the recording is noninvasive and the ECG signal is easy to extract and analyze. HRV describes beat‐to‐beat variation in heart rate (HR) or small differences in RR intervals, thus reflecting the function of the autonomic nervous system (ANS). HRV comprises two components, namely, sympathetic and parasympathetic components. When humans are in a state of heavy mental workload, cardiac activity is controlled mainly by sympathetic nerves, whereas cardiac activity is normally controlled mainly by the vagal nerves (Lean & Shan, 2012). HR increases and the parasympathetic components of HRV decrease in situations of higher mental workload (Lehrer et al., 2010). ECG is one of the earliest physiological methods used to evaluate pilots’ mental workload (Roscoe, 1992). In an early study, HR and HR irregularity (HI) were used to assess mental workload at different flight phases in a simulated flight and were found to differ from phase to phase (Opmeer & Krol, 1973). With the establishment of HRV measurement standards (Task Force of The European Society of Cardiology & The North American Society of Pacing & Electrophysiology, 1996) and the development of acquisition and analysis techniques, ECG remains one of the physiological methods commonly used to evaluate pilots’ mental workload to date (Mansikka et al., 2016a, 2016a). Many recent studies showed that HR and HRV are sensitive to different task demands and can distinguish between levels of mental workload in simulated flights (De Rivecourta et al., 2008; Lehrer et al., 2010; Mansikka et al., 2016a, 2016a) or actual flights (Bonner & Wilson, 2002; Skibniewski et al., 2015; Veltman, 2002; Wilson, 2002). However, some studies showed that HR was not sensitive to mental workload (Lee & Liu, 2003; Wanyan et al., 2014) and others showed the same about HRV (Gentili et al., 2014; Hidalgo‐Muñoz et al., 2018).

Compared with ANS, mental workload is more correlated with the central nervous system (CNS) (Miura et al., 2016). fNIRS is a functional brain imaging method that can be used to evaluate mental workload by assessing CNS activity. Compared with other imaging modalities, it has the advantages of safety, portability, low cost, and high temporal resolution, and it is applicable in procedures involving mobility and interactivity (Boas et al., 2014). It sends out near‐infrared light of 700−900 nm into the cerebral cortex tissue. The near‐infrared light is refracted and absorbed by tissue and then passes through the cerebral cortex. The changes in the hemodynamic indices of the cerebral cortex are then measured by calculating the spectral changes of the near‐infrared light through the cortex. The main chromophores of near‐infrared light in the tissue are oxygenated hemoglobin (HbO) and deoxygenated hemoglobin (HbR) (Ferrari & Quaresima, 2012). Therefore, it can convert the spectral changes to the changes in hemoglobin concentration using the modified version of the Beer‐Lambert law (Boas et al., 2014). The changes in hemoglobin concentration can reflect the metabolism of oxygen in the brain. The prefrontal cortex (PFC) has a functional relationship with mental work, and the allocation of attention resources across multiple tasks has also been associated with activity in the PFC (Mckendrick et al., 2016). Previous fNIRS‐based mental workload studies suggested that fNIRS was sensitive to mental workload (Liu et al., 2017). The fNIRS technology has been used in simulated‐flight‐ and actual‐flight‐based assessments to evaluate mental workload. In a simulated flight study of applying fNIRS, the difficulty of a secondary task was used to manipulate mental workload and a main effect of mental workload with a higher HbO level under high mental workload condition was found (Mouratille et al., 2020). Verdière et al. (2018) used fNIRS connectivity metrics to discriminate between two different landing conditions (manual vs. automated). Besides offline analysis, Gateau et al. (2015) adopted an online fNIRS classifier based on support vector machine to detect working memory load accurately during interaction tasks between a pilot and air traffic control (ATC) within a simulated flight. In addition to simulated flight settings, fNIRS has also been applied in actual flights. Gateau et al. (2018) contrasted changes in the concentrations of oxygenated hemoglobin in PFC between an actual and a simulated flight, and the results showed that pilots in the actual flight condition had higher anterior PFC activation than pilots in the simulator.

These studies suggest that ECG and fNIRS are suitable physiological assessment methods for studying mental workload in flight environments. In some flight phases, pilots need to deal with multiple subtasks at the same time, so multitasking becomes more important. Therefore, it is necessary to evaluate the mental workload of multitasking in a specific flight environment. Hsu et al. (2015) assessed mental workload during National Aeronautics and Space Administration Multi‐Attribute Task Battery, which includes four subtasks: system monitoring, resource management, communication, and tracking. The subtasks were completed at the same time and three levels were set according to the frequency of event occurrence. They found that the low frequency over high frequency ratio (LF/HF) was sensitive to different high mental workload levels. However, this result cannot reflect the respective effect of a subtask because different subtasks have different neurocognitive needs (Stojan & Voelcker‐Rehage, 2021). Stojan and Voelcker‐Rehage (2021) studied the effects of age on brain hemodynamic activity when engaging in three different types of subtasks (stating arguments [ARG], typing numbers [TYPE], and memorizing and recalling information [WM]) while driving. They found that HbO increases and HbR decreases in young drivers during the ARG task, but not during TYPE or WM. To study the influences of specific subtasks on mental workload, a task load gradient needs to be established by controlling the number of subtasks. Puma et al. (2018), for example, elicited mental workload by increasing the number of concurrent subtasks in a multitasking protocol employed in a French airline pilot recruitment process and found that high mental workload can lead to an increase in the theta band of electroencephalographic data. However, to our knowledge, fNIRS has not been studied in a simulated flight multitasking model wherein mental workload is set by increasing the number of subtasks. Our main hypothesis postulates that oxygenation levels in PFC are more activated as the number of subtasks increases during simulated flight multitasking.

To test this hypothesis, the changes in subjects’ PFC hemodynamics during an engaging flight multitasking task were monitored using fNIRS, and PFC activation was discussed. The changes in the NASA‐TLX scores, multitasking performance, mean HR, and HRV were analyzed at the same time. The MATB‐II task is a classical simulated flight multitasking model, but to eliminate the learning effect, the time of substantial training given to the participants in MATB‐II task was more than 80 min (Hsu et al., 2015). The classical psychological task has little learning effects but is quite different from the flight activity. For this purpose, we designed a semiecological multitask model that simulates flight operations abstractly and has smaller learning effects. The model simulates the content of multiple tasks in actual flight such as flight operation, instrument monitoring, and emergency handling.

## 2 METHODS

### 2.1 Participants

Twenty‐six healthy Chinese male undergraduates (mean age, 20.5 ± 0.63 years) participated in the study. None of the participants had previously participated in this task experiment. The Edinburgh Handedness Inventory showed that participants were right‐handed and the average laterality quotient and Decile were 71.67 ± 18.67 and 4.13 ± 3.34, respectively. They had normal or corrected‐to‐normal vision and hearing. All participants read and signed a consent form before the experiment and were later paid for their participation. This research complied with the tenets of the Declaration of Helsinki and was approved by the Institutional Review Board of Air Force Military Medical University.

### 2.2 Equipments and materials

#### 2.2.1 Flight task

**FIGURE 1:**
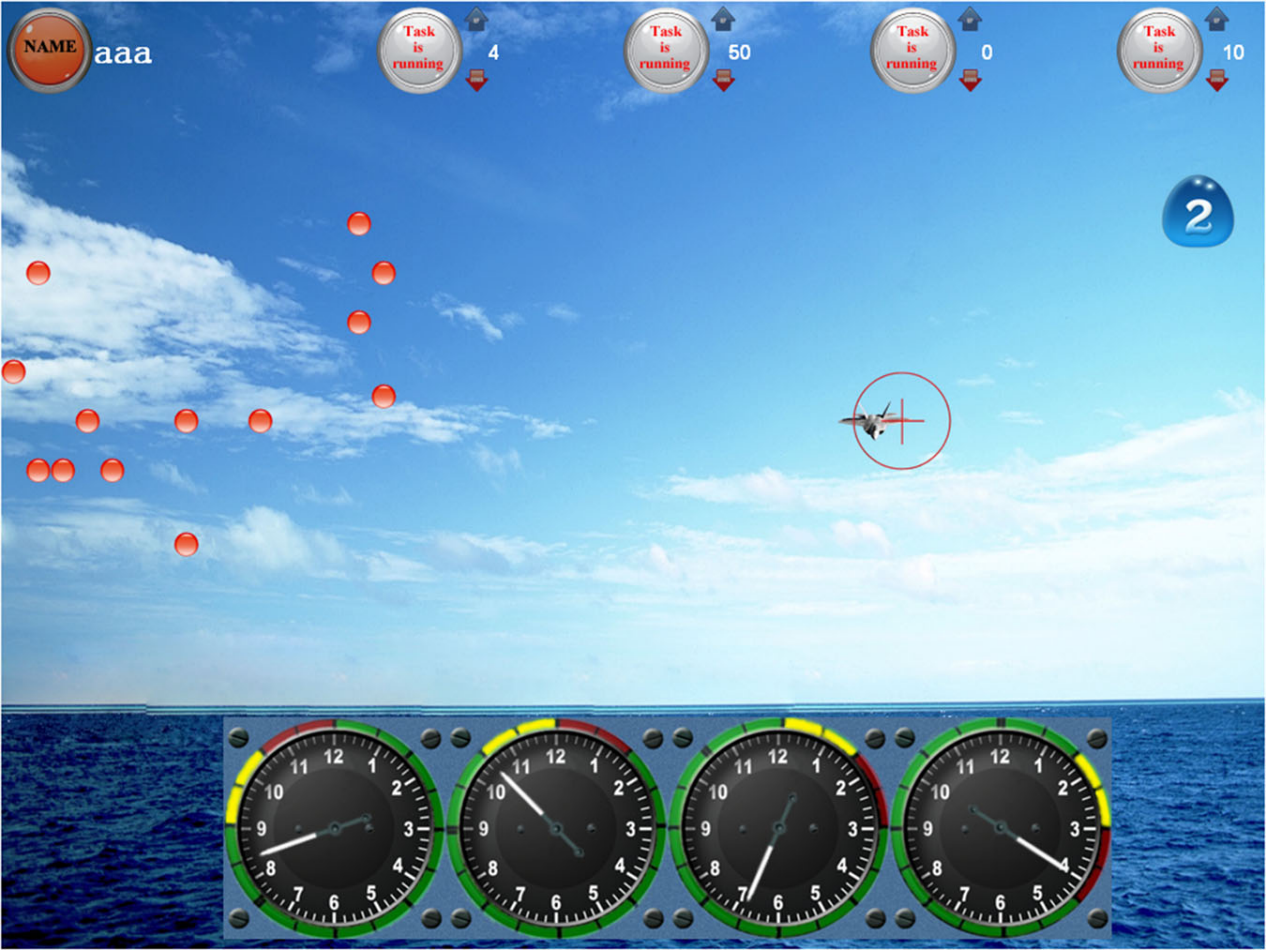
The interface of the multitask model

The task model simulates the multitasking during flight. The multitasking task consists of four subtasks: the flight target tracking task, the meter monitoring task, the emergencies handling task, and the residual capacity task. The residual capacity task is a secondary task, and the other three tasks are primary tasks. The program was run on a computer and the task interface is shown in Figure 1. The upper part of the interface is the task selection area, the middle is the flight target tracking task area, the bottom is the meter monitoring task area, the left side is the emergencies handling task area, and the right side is the residual capacity task area.

#### 2.2.2 Flight target tracking task

Participants were required to track an aircraft‐shaped moving target through a circular cursor by aiming it at the target. The cursor was controlled with a joystick on the right‐hand side. The demands of the task were to aim the circular cursor at the target.

#### 2.2.3 Meter monitoring task

There were four round dashboards in the meter monitoring task area. Each dashboard had a red area defined as a warning area, and the location of each warning area was different. At the beginning of the task, the pointer of each dashboard began to rise clockwise from the bottom at different speeds. Participants were asked to respond by pressing a key when the pointer reached the warning area. Once the subjects responded correctly, the pointer immediately fell back and began to rise clockwise again. If subjects did not respond in time, the pointer continued to rise, until it was over the warning area and then fell back automatically and began to rise clockwise again.

#### 2.2.4 Emergencies handling task

During the task, several red dots appeared sporadically in the emergency handling task area. The number of red dots was a random number between 10 and 20. Participants were asked to count the number of red dots and press the digit of the number. The red dots disappeared once subjects gave the correct response and the next group of red dots appeared 30 s later. If participants had not respond correctly in time, the red dots disappeared automatically 30 s after their appearance, and the next group of red dots would appear.

#### 2.2.5 Residual capacity task

There was always a number in the residual capacity task area during the task. Participants were asked to prioritize primary tasks and then respond by pressing the same number key. The number disappeared once participants gave the correct response, and the next number appeared immediately. If participants did not respond or they responded incorrectly, the number remained until the end of the task.

#### 2.2.6 Subjective ratings

The National Aeronautics and Space Administration‐task load index (NASA‐TLX), developed by NASA in 1988 is a multidimensional mental workload assessment scale (Hart & Staveland, 1988). The NASA‐TLX scale includes six dimensions: MD, mental demand; PD, physical demand; TD, temporal demand; OP, own performance; EF, effort; and FR, frustration. Participants were asked to make a mark on the straight line representing each dimension, which were used as their base scores. Then, the six dimensions were paired, and participants chose the most important dimension of each pair. The weight of each dimension was determined by the times of each dimension chosen. The NASA‐TLX score was calculated from the original score and weight.

#### 2.2.7 Data recording

**FIGURE 2:**
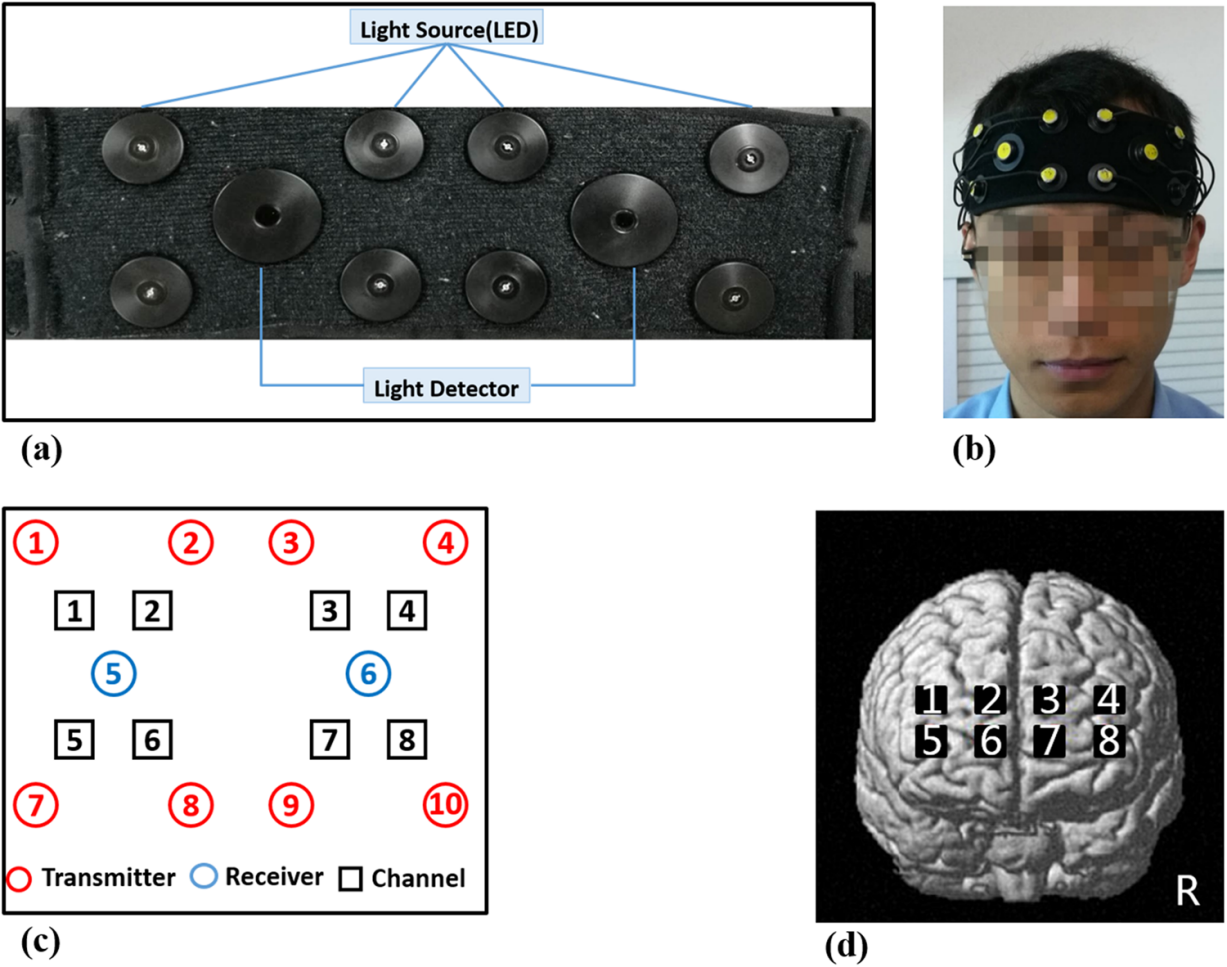
The OctaMon fNIRS system: (a) the sensor with eight light sources and two detectors and eight channels. (b) The position of the sensor on participants' head; (c) the standard configuration of OctaMon; (d) eight channels on brain surface image

An OctaMon (Artinis Medical Systems B.V, Netherlands) continuous‐wave fNIRS system (see Figure 2) was used to record prefrontal fNIRS signals. This device was an eight‐channel NIRS device and recorded at a 2 Hz sampling rate. The sensor included eight LED light sources (transmitter) that emit light at 760 and 850 nm wavelength light and two detectors (receiver). The system could distinguish the light sources, and the principle is called the time‐sequenced principle. The distance between the light sources and detectors was 3.5 cm.

A chest band (Institute of Aerospace Medicine, China) was used to record ECG signals. It had three electrodes: the positive was at the fifth rib in the left anterior axillary line, the negative in the right anterior axillary line, and the reference 3 cm to the right of the positive electrode. Signals were recorded using a data‐recording box.

### 2.3 Procedure

Before the experiment, participants were introduced to the content and process of the experiment and were then asked to read and sign the informed consent form. Participants read the description of the Edinburgh Handedness Inventory scale and completed the scale. They then went through a practice round of the multitasking task, which lasted about 10 min. During the practice, the participants’ operations were observed to ensure that they learned how to complete the task. After the practice, the participants wore the fNIRS device head band and ECG device chest band. Care was taken that the light sources and the detectors were completely touching the forehead skin, and hair from the eyebrows or side of the head was kept away from the sources and detectors.

Participants rested for 1 min and then began the multitasking task. Three multitasking tasks of different task load levels need to be completed. In the low load condition phase, participants were asked to accomplish the flight target tracking and residual capacity tasks simultaneously. In the medium load condition phase, they were asked to accomplish the other three tasks simultaneously, excluding the emergencies handling task. In the high load condition phase, they were asked to accomplish four tasks at the same time. Each task lasted 180 s, and the sequence was counterbalanced among the participants. After the completion of each multitasking task, participants completed the NASA‐TLX scale and rested for 1 min. After completing the last multitasking task, the head and chest bands were removed and the experiment was complete. The experiment lasted approximately 1 h.

### 2.4 Statistical analysis

Raw fNIRS data (8 channels × 2 wavelengths) were preprocessed using NIRS‐SPM4 software, an SPM and MATLAB‐based software package for statistical analysis of near‐infrared spectroscopy (NIRS) signals. Statistical analysis of the NIRS data used a mass‐univariate approach based on the general linear model (GLM). Raw data were smoothed using a wavelet transform, which can attenuate high frequency noise components. A wavelet transform was applied to decompose NIRS measurements of hemodynamic signals and uncorrelated noise components at distinct scales. The hemodynamic response function as a low‐pass filter was used to reduce high‐frequency physiological noise such as heartbeats. We assessed the degree of PFC activation for each task using a GLM approach and acquired β‐coefficients of HbO signals for each condition and within each channel. The sign and magnitude of each β‐coefficient indicated the direction and intensity of the change in blood oxygen levels during each condition. Although we only used HbO for analysis, NIRS‐SPM accurately localized the HbO and HbR signals onto the cerebral cortex in the spatial preprocess, and the HbO signal has a higher signal‐to‐noise ratio, which ensured that we obtained valid activations (Sagiv et al., 2019; Ye et al., 2009).

From the recorded ECG signals, the following features were calculated using the software of the chest band (Institute of Aerospace Medicine): the mean value of the HR (mean HR), standard deviation of the RR intervals (SDNN), root mean square of the successive difference of the RR intervals (RMSSD), and LF/HF.

One‐way repeated measures analysis of variance (ANOVA) with within‐participants’ factors and task load were carried out to test whether the effects of task load were statistically significant on the different measures (scores of NASA‐TLX, performance of multitasking tasks, oxygenation activation levels, HR, and HRV). Statistical analysis was conducted using SPSS software, version 18.0. Post‐hoc testing was conducted with Bonferroni. Pearson correlations were used between different indices. ECG internal indices (i.e., HR and HRV) and performance internal indices (i.e., average distance and meter response time) were not performed for correlation analysis. A 5% significance level was adopted in all tests and Bonferroni correction was used for multiple comparison.

## 3 RESULTS

### 3.1 Subjective load

**FIGURE 3:**
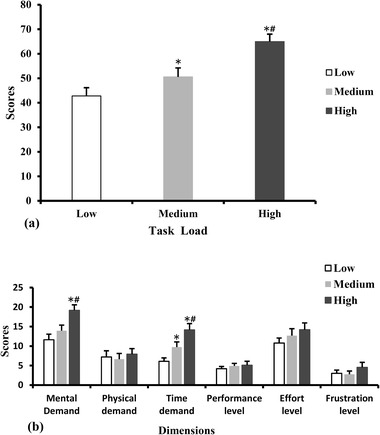
NASA‐TLX scores scale in different task load conditions: (a) total scores, (b) scores of six dimensions. *p < .016, as compared with low task load; ^#^ *p *< .016, as compared with medium task load. The error bars represent the standard error of the mean

One‐way repeated measures ANOVA conducted on NASA‐TLX scores revealed significant main effects of task load [*F* (2, 26) = 56.96, *p* < .001]. High task load had the highest NASA‐TLX score (*p *< .016 with Bonferroni correction for multiple testing). Figure 3a shows the total scores of NASA‐TLX for the three different multitasking tasks.

The scores of each dimension are shown in Figure 3b. The effects of task load on mental demand [*F* (2, 26) = 17.04, *p* < .001] and time demand [*F* (2, 26) = 24.19, *p* < .001] were significant. High task load had a higher score for mental demand than low task load and medium task load (*p *< .016 with Bonferroni correction for multiple testing). The scores of time demand increased gradually with the increase in task load (*p *< .016 with Bonferroni correction for multiple testing).

### 3.2 Behavioral performance

Table 1 shows the behavioral performance of the three task loads. The performance of the flight target tracking task included the average tracking distance and the number of alarms. The tracking distance was the distance between the circular cursor and target. When the distance was larger than 30 mm, the computer recorded an alarm. The average distance [*F* (2, 26) = 17.60, *p* <.001] and the number of alarms [*F* (2, 26) = 32.77, *p* < .001] in the three different tasks were significantly different. Post‐hoc tests with Bonferroni showed that the average distance and number of alarms increased gradually with the increase in task load (*p* < .016 with Bonferroni correction for multiple testing).

**TABLE 1:** Behavioral performance in different task load conditions ($\bar{x}\pm s$)

Feature	Low task load	Medium task load	High task load
Average distance (mm)	21.67 ± 3.71	23.48 ± 4.86*	26.07 ± 6.50*, ^#^
Number of alarms	71.81 ± 36.00	91.92 ± 45.87*	116.23 ± 50.27*, ^#^
Number of numeral response	104.00 ± 31.68	76.62 ± 35.99*	40.58 ± 30.89*, ^#^
Meter response time(s)	none	3.37 ± 1.26	3.57 ± 1.58
Meter response accuracy (%)	none	83.92 ± 21.70	82.30 ± 22.92
Emergency response time(s)	none	none	18.16 ± 4.63
Emergency response accuracy (%)	none	none	80.20 ± 18.46

* *p* < .016, as compared with low task load.

^#^ *p *< .016, as compared with medium task load.

The number of numeral response of the residual capacity task were significantly different [*F* (2, 26) = 120.47, *p* < .001]. The response times decreased gradually with the increase in task load (*p* < .016 with Bonferroni correction for multiple testing). The response time [*F* (1, 26) = 0.95, *p* > .05] and accuracy [*F* (1, 26) = 0.43, *p* >.05] of the meter monitoring task was not significantly different. The average accuracy and response time of the emergencies handling task in high task load were 80.20 ± 18.46% and 18.16 ± 4.63 s, respectively.

### 3.3 PFC activation

**FIGURE 4:**
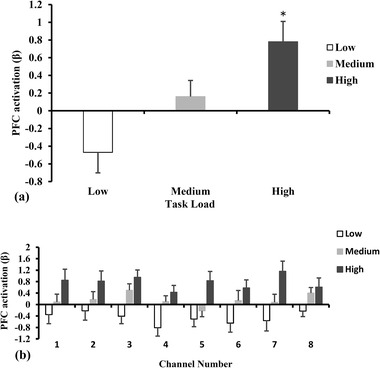
PFC activation in different task load conditions. *p < .016, as compared with low task load. The error bars represent the standard error of the mean

The PFC activation (β) results in different multitasking tasks are shown in Figure 4. The effects of task load on PFC activation (Figure 4a) were significant [*F* (2, 26) = 8.04, *p* < .001]. High task load had higher PFC activation than low task load (*p* < .016 with Bonferroni correction for multiple testing). Figure 4b shows the average PFC activation of each channel in different task loads. The effects of task load on PFC activation of channel 1[*F* (2, 26) = 3.50, *p* = .038], channel 3[*F* (2, 26) = 7.07, *p* = .002], channel 4 [*F* (2, 26) = 6.32, *p* = .004], channel 5 [*F* (2, 26) = 6.62, *p* =.003], channel 6 [*F* (2, 26) = 4.28, *p* = .019], channel 7 [*F* (2, 26) = 6.14, *p* = .004], and channel 8 [*F* (2, 26) = 3.70, *p* = .032] were significant.

### 3.4 Heart rate variability

Table 2 shows the HR and HRV in different tasks. The effects of task load on SDNN [*F* (2, 26) = 0.13, *p* = .875], RMSSD [*F* (2, 26) = .18, *p* = .840], and LF/HF [*F* (2, 26) = 0.52, *p* = .595] were not significant. However, the effects of task load on mean HR were significant [*F* (2, 26) = 6.27, *p* = .004]. While post‐hoc tests showed no significant HR differences between each task load after *p*‐value correction, HR was higher during high task load than low task load with uncorrected *p*‐values at α = .05.

**TABLE 2:** Heart rate and heart rate variability in different task load conditions ($\bar{x}\pm s$)

Feature	Low task load	Medium task load	High task load
Mean HR (beat/min)	67.15 ± 9.35	67.96 ± 10.53	69.58 ± 12.50
SDNN	48.12 ± 13.00	48.85 ± 11.11	49.21 ± 14.85
RMSSD	47.10 ± 20.80	46.23 ± 19.50	46.99 ± 23.03
LF/HF	1.36 ± 1.19	1.41 ± 1.26	1.56 ± 1.02

Abbreviations: LF/HF, ratio of the low frequency over the high frequency; RMSSD, root mean square of the successive difference of the RR intervals; SDNN, standard deviation of the RR intervals.

### 3.5 Correlation analysis

**FIGURE 5:**
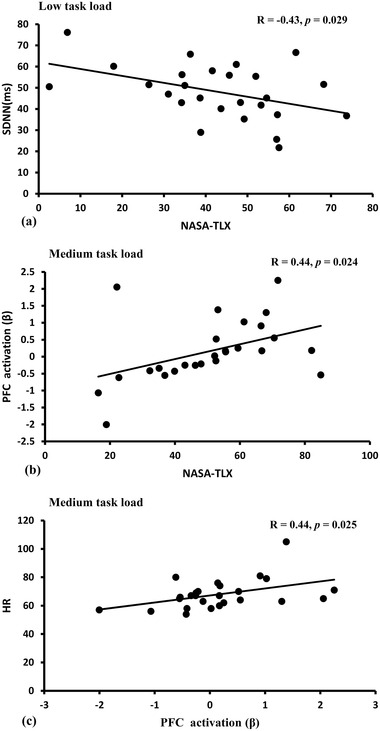
The scatterplot with regression lines

The analysis showed significant correlations in three tests. Figure 5a shows a negative correlation between SDNN and NASA‐TLX in low task load [R (26) = −0.43, *p* = .029]: the higher subjective mental workload was, the smaller the SDNN became. Figure 5b shows a positive correlation between brain activation and NASA‐TLX in medium task load [R (26) = 0.44, *p* = .024]: the higher subjective mental workload was, the more the PFC was activated. Figure 5c shows a positive correlation between brain activation and HR in medium task load [R (26) = 0.44, *p* = .025]: the higher the PFC activation was, the higher HR became. None of the other correlations were significant (R < 0.40 and *p* > .05 in all cases).

## 4 DISCUSSION

The purpose of this experiment was to use fNIRS and ECG to measure the functional activation of the PFC and HR variability to evaluate the mental workload of multitasking during a simulated flight. The program simulated flight characteristics such as target tracking, meter monitoring, and emergency handling. The difficulty of the multitasking tasks was set by the number of subtasks completed at the same time.

NASA‐TLX scores increased as the number of subtasks increased, indicating that different levels of cognitive demand in multitasking tasks were successfully elicited. As one of the most used subjective assessment scales, NASA‐TLX can reflect mental workload from six dimensions, so it can diagnose the source of mental workload (Rubio et al., 2004). In this experiment, the differences in NASA‐TLX scores in different tasks were reflected in mental demand and temporal demand. This indicated that the high mental workload of participants was mainly a result of time pressure, which was different from the task model that said mental workload resulted from difficulty of operation or memory (Fallahi et al., 2016). In the experiment, there was no significant difference in subjective effort or frustration with the increase in task difficulty. This may be because the participants had a subjective feeling that they had completely paid the remaining cognitive resources to the secondary task after completing the primary tasks.

As indicated by an increase in the average tracking distance and number of alarms, the performance of the flight target tracking worsened as the number of subtasks increased. It showed that other subtasks affected the tracking task performance. Other subtasks took up the participants’ cognitive resources, and the cognitive resources available to the tracking task were reduced, leading to a decline in their tracking task performance. However, the performance of the meter monitoring task was not affected by the emergency handling task. This may be because subjects had a resource allocation policy when completing the emergencies handling task. The resource allocation policy is a propensity that is adopted by the performer regarding which task was favored (Wickens, 2002). The average reaction time of the emergency handling task was 18.16 s. The long response time indicated that subjects chose to prioritize the meter monitoring task over to the emergencies handling task. In fact, emergency handling is often prioritized over meter monitoring in real‐world situations. However, multiple task demands may influence operators’ strategies, such as delaying priority tasks (Wickens et al., 2016). Reversed priorities, for example, prioritizing ATC communications over maintaining flight stability can lead to an accident (Schutte & Trujillo, 1996). As a secondary task, the residual capacity task performance had the same result as that of the flight target tracking task. The theoretical basis of the secondary task was the operator's limited attention resources, which can be used to assess the mental workload associated with simulated flight or driving (Heine et al., 2017). In the experiment, participants were asked to complete the primary tasks preferentially and complete the secondary task with their remaining cognitive resources. As the task load increased, their residual capacity decreased, leading to a decrease in the number of numeral response in the secondary task. This is consistent with Wicken et al. (2016) that the higher mental workload is, the less residual capacity from the primary task there is available for the secondary task, which leads to a worse secondary task performance.

fNIRS has been widely used in the field of brain research including cerebral structure and function research, brain‐computer interface, adaptive interface, mental workload assessment, etc. (Boas et al., 2014; Pan & Jiao, 2013). fNIRS is sensitive to changes in mental workload (Liu et al., 2017; Mouratille et al., 2020). Changes in task difficulty, including difficulty with memory or information processing load, can lead to changes in the activation degree in relevant brain regions (Foy et al., 2016). Brain areas sensitive to mental workload have been shown to elicit activations during time‐limited cognitive activities (Barch et al., 1997). In this experiment, with the increase in the number of subtasks, the information load increased and the PFC activation increased. These findings were similar to those of previous studies, showing that the PFC was particularly sensitive to mental workload (Ayaz et al., 2012). As a collection of interconnected neocortical areas, PFC is mainly related to high cognitive functions such as working memory, decision making, executive control (Mckendrick et al., 2016). According to the location of the regions, PFC can be divided into the rostral PFC, dorsolateral PFC, ventrolateral PFC, medial PFC, and orbitofrontal PFC (Miller & Cohen, 2001). Although different regions of PFC have different and specific functions (Miller, 2000), mental workload, especially in flight, influence selective attention, spatial attention, episodic memory, cognitive control, task switching, attention allocating, and decision‐making (Chenot et al., 2021), which need close and interactive relationship of different regions of PFC. Therefore, the activation of the whole PFC—instead only specific areas—was usually used for assessing mental workload (Causse et al., 2017; Causse et al., 2019; Gateau et al., 2015). The results of this experiment showed that PFC activation can be used to evaluate the mental workload caused by the number of subtasks in multitasking tasks. The theoretical basis is the multiple resources model. According to Wickens (2008), the multiple resources model has four dimensions (processing stages, perceptual modalities, visual channels, and processing codes) and each dimension has separate and distinct pools of attentional resources. Tasks completed at the same time received resources from the same attentional resources pool, which generated resource competition. In the multitasking task, each subtask needed the resources of focal vision and the competition of cognitive resources existed between each subtask. Therefore, the increase in subtasks can mobilize more cognitive resources of subjects, which may lead to an increase in the activation of the PFC. Further work is required to investigate whether activations observed in the PFC were induced sustainably or transiently.

SDNN and RMSSD are time‐domain features of HRV. SDNN is treated as a reflection of the overall autonomic nervous system function while RMSSD is treated as a reflection of the parasympathetic function (Malik, 1996). As frequency domain features of HRV, LF is mainly associated with sympathetic activity and HF is associated with parasympathetic activity (Kamath & Fallen, 1993). When mental workload increased, sympathetic activities increased while parasympathetic activities decreased (Fallahi et al., 2016). Regrettably, sensibility of HRV and HR to assess mental workload in flight was not consistent as we introduced above. In this experiment, there were no significant differences in HRV among different multitasking tasks, but the mean HR increased with the increase in mental workload, which was consistent with some simulated flight studies (e.g., Gentili et al., 2014; Hidalgo‐Muñoz et al., 2018). A possible explanation for these changes is that HRV is less sensitive in certain practical circumstances (De Rivecourta et al., 2008). De Rivecourta et al. (2008) indicated that HRV is less sensitive to measuring gradual changes in task demands at relatively high mental workload levels. In this study, multitasking required the completion of at least two subtasks at the same time, so the mental workload is relatively high. As was done in Hidalgo‐Muñoz et al.’s (2018) study, a secondary task was added to the program and consistent results were obtained. Some studies indicated that although mental effort affected both HR and HRV, the effects on HR were more robust (Veltman, 2002).

PFC activation at high task load was higher than PFC activation at low task load in the post‐hoc analysis, while no significant differences of PFC activation and HR between the other contrasts were found. This could be explained by theory of reserve capacity (Wickens et al., 2016). In this study, the subjects were required to use all their remaining capacity to complete the secondary task so as to maintain performance in that task. In fact, an increase in task load can still generate more cognitive resources, but the participants used up their mental effort subjectively during each multitasking task, which was reflected by the subjective effort scores. Therefore, gradual changes in the generated cognitive resources shrink during different multitasking. PFC activation and indices of ECG were not correlated to task performance in the study. This dissociation between performance and nervous system activity was also found by other studies (Ayaz et al., 2012; Causse et al., 2017) and can be explained by the neural efficiency hypothesis of intelligence, which states that intelligent individuals show higher brain activation efficiency when performing cognitive tasks (Neubauer & Fink, 2009). However, SDNN was negatively correlated with subjective mental workload in the low task load condition, while PFC activation was positively correlated with HR and subjective mental workload in the medium task load condition. Correlational relationships were not found in the high task load condition. That may be related to a plateau in high task load, which represents a processing limit (Causse et al., 2019); that is, physiological indices can predict subjective mental workload across participant to a certain extent. In addition, the PFC activation and HR showed effects on task load, suggesting that combining fNIRS and ECG to evaluate mental workload during multitasking in flight may be an effective approach. Of course, future studies on in‐flight multitasking should explore more advanced methods of fNIRS, such as wavelet coherence analysis and methods of graph theory, which could provide a rich picture of large scale distributed neural sources (Leff et al., 2015).

## 5 CONCLUSIONS

In this study, we found that task performance decreased with increasing mental workload during multitasking. The increased mental workload was primarily due to time pressure. HR and the PFC activation can be used to detect changes in mental workload during multitasking in simulated flight. These results can provide an effective reference for evaluating or quantifying the mental workload of pilots during multitasking. Future work should be carried out to provide a criterion for evaluating mental workload.

## CONFLICT OF INTEREST

The authors have no conflict of interest to declare.

### PEER REVIEW

The peer review history for this article is available at https://publons.com/publon/10.1002/brb3.2489

## Data Availability

The data of this study are available from the corresponding author on reasonable request.
